# Results of a Five-Year Experience in First Trimester Preeclampsia Screening

**DOI:** 10.3390/jcm11154555

**Published:** 2022-08-04

**Authors:** Stella Capriglione, Ferdinando Antonio Gulino, Silvia Latella, Giovanna De Felice, Maurizio Filippini, Miriam Farinelli, Francesco Giuseppe Martire, Elsa Viora

**Affiliations:** 1 Department of Obstetrics and Gynecology, Ospedale “Santa Maria Alla Gruccia” Piazza del Volontariato 2, 52025 Montevarchi, Italy; 2 Department of Obstetrics and Gynecology, Azienda di Rilievo Nazionale e Alta Specializzazione (ARNAS) Garibaldi, Via Palermo 636, 95126 Catania, Italy; 3 Department of Obstetrics and Gynecology, Istituto per la Sicurezza Sociale, 47893 Cailungo, Borgo Maggiore, San Marino; 4 Gynecological Unit, Department of Surgical Sciences, University of Rome “Tor Vergata”, 00133 Roma, Italy; 5 Obstetrical-Gynecological Ultrasound Unit, Department of Obstetrics and Gynecology, Sant’Anna Hospital, 10126 Turin, Italy

**Keywords:** preeclampsia, first trimester, screening, fetal DNA

## Abstract

Background and Objectives: The study aimed to evaluate the ability defining the risk of developing preeclampsia by a screening test carried out in the first trimester (between 11 + 0 and 13 + 6 weeks of gestational age), in order to identify high-risk women requiring more intensive health surveillance. The secondary objective was to evaluate the ability of this test to predict the risk of adverse obstetric outcomes such as fetal growth restriction, intrauterine fetal death, gestational hypertension, HELLP syndrome, placental abruption, and preterm birth. Materials and Methods: This was a single-center study, conducted at the Operative Unit of Obstetrics of the State Hospital of the Republic of San Marino. Medical history was collected at the time of enrolment in writing. Subsequently, obstetric outcomes were collected for each enrolled woman, through the analysis of medical records. Results: From October 2014 to May 2019, 589 pregnant women were recruited, of whom, 474 (80.5%) were included in the “low-risk” group, and 115 (19.5%) in the “high-risk” group. At the time of analysis of this population, the obstetric outcomes were available for 498 women (84.5%), while 91 cases (15.5%) were current pregnancies. The PI of the uterine arteries was not significantly different between the two study groups. Otherwise, a significant difference was highlighted for MAP, which is higher in the case of pregnancies at high risk based on the risk factors only, and for PAPP-A, higher in the case of low-risk pregnancies. Regarding the percentage of fetal DNA, according to the most recent literature data, in our series, we report a statistically significant difference of the average between the low and high-risk groups. Conclusions: In our study, we demonstrate that the multiparametric screening test for early PE performed well in identifying women at high risk of early PE, which certainly has the most severe maternal–fetal outcomes. The data reported that ASA intake at low doses is significantly higher in the population with high-risk tests for both early PE and late PE suggest once again that anamnestic evaluation plays an essential role in women’s screening.

## 1. Introduction

Current guidelines from the Italian Association of Preeclampsia (AIPE) for the early identification of women with a higher risk of developing preeclampsia (PE) are mainly based on maternal personal, medical, and family history [1]. Among these risk factors, the main role is played by [2,3,4,5,6,7,8,9,10,11,12]:

- Nulliparity;
- Afro-Caribbean ethnicity;
- Maternal age < 20 or >35 years;
- Maternal body mass index (BMI) > 30;
- Chronic maternal hypertension;
- Diabetes, renal pathologies, and autoimmune diseases such as scleroderma, rheumatoid arthritis, and systemic lupus erythematosus;
- Positive obstetric history of PE;
- Positive familiar history of PE.

Early-onset preeclampsia is usually defined as preeclampsia that develops before 34 weeks of gestation, whereas late-onset preeclampsia develops at or after 34 weeks of gestation.

We aimed to evaluate the ability to define the risk of developing preeclampsia by a screening test carried out in the first trimester (between 11 + 0 and 13 + 6 weeks of gestational age). The calculation of the risk is the result of the association of risk factors such as nulliparity, Afro-Caribbean ethnicity, BMI ≥ 30, chronic maternal hypertension, obstetric history, positive family history of PE, positive obstetric history of intrauterine fetal death (IUFD), or intrauterine growth restriction (IUGR), serum levels of PAPP-A (Pregnancy-Associated Plasma Protein A) and PlGF (Placental Growth Factor), ultrasound measurement of the pulsatility index of uterine arteries (PI), and mean arterial pressure (MAP) [13,14,15,16,17,18,19,20,21,22,23,24,25,26,27,28].

This test would identify pregnancies at higher risk of preeclampsia even in a population of pregnant women with no risk factors. Therefore, we can identify women requiring more intensive health surveillance, starting from the first weeks of pregnancy [29], with the chance to adopt prophylactic strategies, such as the daily intake of low-dose acetylsalicylic acid, which are more successful if initiated before the end of the placentation process, that is abnormal in the case of PE [30,31,32,33]. As a secondary objective, we evaluated the test’s ability to predict the risk of developing adverse obstetric outcomes such as fetal growth restriction (FGR or IUGR), intrauterine fetal death (IUFD), gestational hypertension, HELLP syndrome, placental abruption, and preterm birth.

## 2. Materials and Methods

This was a single-center study, conducted at the Operative Unit of Obstetrics of the State Hospital of the Republic of San Marino. Women were selected according to the following inclusion criteria:

- Maternal age > 18 years;
- Singleton pregnancy;
- Gestational age between 11 + 0 and 13 + 6 weeks;
- CRL (Crown Rump Length) between 45 and 80 mm;
- Signed informed consent.

At the time of enrollment, the women were divided into two groups:

(1) “High-risk” group
  Including pregnant women having one of the following risk factors:
  - BMI ≥ 30 kg/m^2^
  - Presence of Chronic Arterial Hypertension;
  - Previous history of preeclampsia;
  - Previous history of intrauterine fetal death (IUFD) related to placental vascular disease (PVD), or Intrauterine Growth Restriction (IUGR).

(2) “Low-risk” group

This group included all women without the risk factors mentioned above.

The aims and the methodology of the study were explained appropriately to the women and informed consent was obtained in all included patients.

Personal, familial, medical, and obstetric history was collected. Systolic, diastolic, and mean arterial blood pressure values were assessed. CRL and uterine artery PI were assessed by 2D and Doppler ultrasound. A venous blood sample was obtained for the dosage of PAPP-A and PlGF markers and from January 2019, a blood test for fetal DNA was also carried out for free, and the results for the women recruited before January 2019 who had independently undergone fetal DNA testing were also collected.

The medical history of the recruited women included: maternal age, parity, method of conception, ethnicity, tobacco smoke in pregnancy, alcohol or drugs abuse in pregnancy, gestational age calculated based on last menstrual cycle (LMP), chronic hypertension, diabetes mellitus, chronic intake of medical treatment such as antihypertensives, acetylsalicylic acid, and insulin. Bodyweight and height were measured if women were unable to provide this information, and BMI was calculated.

Obstetric history was obtained focusing on previous episodes of preeclampsia in multiparous women, previous cases of IUGR, previous IUFD, previous miscarriages, previous terminations, and other complications arising in previous pregnancies.

Family history of preeclampsia was investigated by asking for information regarding the course of pregnancy of the mother and any sister.

The day of first trimester screening, measurements of systolic blood pressure (SBP) and diastolic blood pressure (DBP) were performed using an automatic and electronic sphygmomanometer, in a sitting position. The measurement was repeated 4 times, 2 in the left arm and 2 in the right arm, alternating the measurement side. All measurements were noted and the mean arterial pressure (MAP) was calculated taking into account the higher systolic and diastolic values, using the following equation: MAP = DBP + 1/3 (SBP-DBP).

Regarding MAP, its measurement in the first trimester was found to be more predictive of preeclampsia onset risk, compared to systolic and diastolic blood pressure [2].

A single venous blood sample was obtained for the dosage of PAPP-A and PlGF markers and the samples were sent to the Modena Test Analysis Laboratory.

Measurements of the fetus (Crown Rump Length (CRL) and Nuchal Translucency (NT)) and uterine arteries pulsatility index (PI) were evaluated during the first trimester ultrasound screening at 11 + 0–13 + 6 weeks’ gestational age.

CRL and NT ultrasound measurements were performed according to the criteria suggested by the Fetal Medicine Foundation (FMF). 

The identification of the uterine arteries was obtained using a Voluson E8 device (GE Healthcare; Zipf, Austria) with a transabdominal multifrequency volumetric probe looking for a sagittal section of the uterine cervix and the inferior uterine segment, adjacent to it. The endocervix is identifiable at the junction level between these two structures. Uterine arteries are detectable in the vascular plexus of the paracervical tissue at the endocervix level. They can be identified by color Doppler at the point where they deviate in the cranial direction before ascending towards the uterine body and branching in the arched arteries.

To obtain a good and repeatable measurement for the pulsatility index (PI) of the uterine arteries (UA), the Doppler gate must be set to the size of 2 mm and the probe insonation angle must vary by less than 30° in relation to the longitudinal axis of the uterine artery. In addition, at least 3 waves with a similar shape must be displayed on the graph. In the fluximetric wave profile, the presence of protodiastolic measures (protodiastolic NOTCH) was evaluated, and indexed for reduced compliance of the uteroplacental circle and intervillous spaces.

The collection of clinical–anamnestic data was performed at the time of enrollment in writing. The data were subsequently collected on prospective database, for analysis that took place anonymously.

Subsequently, obstetric outcomes were collected for each enrolled woman, through the analysis of medical records, evaluating:

- Gestational age at birth;
- Neonatal weight at delivery;
- Apgar at 10 min;
- Mortality (≥23 + 0 weeks) and miscarriage (<22 + 6 weeks);
- Development of gestational hypertension, gestational diabetes, preeclampsia, IUGR, HELLP syndrome, placental abruption, eclampsia;
- Type of labor and possible indication for induction, type of birth, and possible indication for caesarean section.

All continuous variables were reported as mean ± standard deviation (SD), while the categorical variables were reported as frequency or percentage.

The level of biochemical markers (PAPP-A and PlGF) was evaluated by the parametric test (*t*-test) with statistical software SPSS version 23, whose level of statistical significance was established for a *p* < 0.05.

The data collection for each woman was carried out through the Software and the algorithm provided by the Fetal Medicine Foundation “PreEclampsia Predictor”, which allows us to calculate the risk of developing preeclampsia according to the method we investigated. It was taken into account, in addition to the risk factors from the history, the serum level of PlGF and PAPP-A, the calculated value of MAP, and the measured values of the uterine arteries PI. The cut-off was set at 1:20.

Then, we started to investigate other adverse outcomes: gestational hypertension, HELLP syndrome, placental abruption, *p*-PROM, gestational diabetes, miscarriage rate, cholestasis, IUGR, intrauterine fetal death, preterm delivery, Apgar < 7 at 10′, “small for gestational age” (SGA), and “large for gestational age” (LGA) newborn.

These were considered both individually and divided into the categories “maternal composite adverse outcomes” and “fetal composite adverse outcomes”, to evaluate the predictive ability of this method of screening for the risk of their occurrence. Similarly, the type of labor and the type of delivery were evaluated.

Regarding fetal DNA, data were collected from January 2019 for all women because the test was provided by the national health system. Previously we collected the data of those women who had performed it privately. We recorded the outcome and percentage of fetal DNA isolated in a maternal blood sample.

## 3. Results

**Figure 1:**
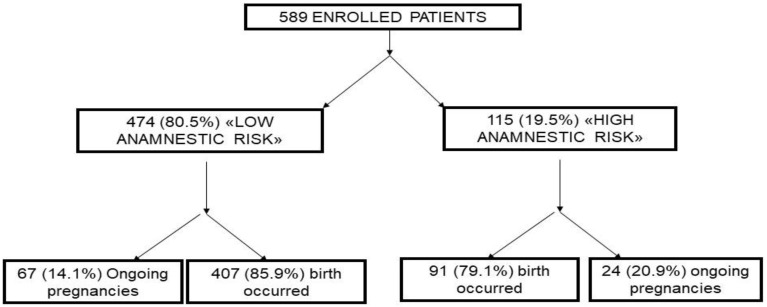
Flow-chart of the study.

From October 2014 to May 2019, 589 pregnant women were recruited, of whom, 474 (80.5%) were included in the “low-risk” group, and 115 (19.5%) in the “high-risk” group (Figure 1).

At the time of analysis of this population, the obstetric outcomes were available for 498 women (84.5%), while 91 cases (15.5%) were ongoing pregnancies. The mean age of the population was 33 years (DS ± 6.2) without statistically significant differences in the two groups. The most represented area of maternal origin was Caucasian. No statistically significant differences were found regarding the “ethnic” variable between the two study groups.

Parity was greater in women belonging to the “high-risk” group than in the “low-risk” group (*p* = 0.013). There was no statistically significant difference in the method of conception between the two groups. For each pregnant woman, the BMI was calculated at the time of enrollment. The incidence of obesity (BMI > 30) is significantly higher in the “high-risk” population (*p* = 0.02) as this is one of the inclusion criteria in the sub-population defined as “high risk”. Seven women were affected by mellitus diabetes before the onset of the pregnancy. The difference between the incidence of this pathology in the two study groups is not statistically significant. A total of 4.2% of the study population claimed to continue to smoke during pregnancy and these women are equally represented in the two groups. All the above data are reported in Table 1.

Table 2 shows the biochemical and biophysical characteristics of the study population. The blood pressure values recorded at the time of enrolment were higher in the population defined as “high risk”. Systolic blood pressure had a mean value of 136 mmHg in this group versus a value of 110 mmHg in the low-risk women. Diastolic had a mean value of 81 mmHg in the “high-risk” group and 74 mmHg in the “low-risk” group. Consequently, the mean MAP values (mean arterial pressure) were higher in the “high risk” population, with a value of 96.2 mmHg (DS ± 9.4) compared to the value of 81.8 mmHg (DS ± 7.1) for the “low-risk” population (*p* = 0.032). Among the PI (pulsatility index) values of the uterine arteries, measured bilaterally at enrollment, we considered the highest one between the two uterine arteries, and no statistically significant differences were found between the two study groups.

The mean PAPP-A value is significantly lower in the “high-risk” group (*p* = 0.002). However, there are no significant differences between the mean values of PlGF in the two groups. Obesity (BMI ≥ 30), chronic hypertension and a history of preeclampsia, fetal endouterine death, and intrauterine growth restriction are exclusively represented in the “high-risk” group as they represent the inclusion criteria for this group. The incidence of the above-cited characteristics in the study population is described in Table 3.

At the time of data analysis, 498 women completed their pregnancy, corresponding to 84.5% of those enrolled. A total of 407 women belonged to the “low-risk” group, while 91 were “high risk”. In all, 15.3% of women took low-dose acetylsalicylic acid daily during pregnancy: the intake was statistically higher in the “high-risk” population, an indication of the presence of more risk factors for placental vascular disease in these women. The incidence is described in Table 4. Analyzing the pregnancy outcome, among these 498 cases, the difference between the two study groups was significant regarding the incidence of maternal adverse outcomes, which were significantly more frequent in the “high-risk” group (OR = 4; 95% CI 2.4–8.1). The incidence of fetal complications is not statistically significant.

In the analyzed population, we found 12 cases of miscarriage, 9 in the “low-risk” group and 3 in the “high-risk” group. The difference between the two populations regarding the incidence of gestational hypertension is not statistically significant.

Only six cases of preeclampsia were recorded and they occurred in the group of pregnant women defined as “low risk”. One of the cases of preeclampsia was in a 30-year-old nulliparous woman, at 28 weeks of gestation, who had not taken ASA and had a BMI of 21 and is the same woman who developed the only case of intrauterine growth restriction (IUGR).

Hypertensive disorders affected 5.6% of the population in our study, with an incidence not significantly different in the two groups. There were no cases of HELLP Syndrome, placental abruption, intrauterine fetal death, and Apgar < 7 at 10′ at birth. Preterm delivery had an incidence that was not significantly different in the two groups.

A total of 83 women (17%) developed gestational diabetes. 48 cases (12%) were “low risk”, all on diet alone, while the other 35 (38.8%) cases were in the “high-risk” group. Treatment with diet therapy was sufficient in 78 cases, while insulin therapy was required in 5 cases. The difference between the two groups is statistically significantly different (OR = 8.1; IC 95% 2.1–27.4). The difference between the populations in the type of labor was significant (OR = 4.1; 95% CI 0.8–8.7). The number of induced labors was higher in the “high-risk” group.

The difference between the type of delivery was not significantly different between the two populations. The difference between the LGA (large for gestational age > 90th centile) in the two group results were statistically significant (OR = 4.9; CI 95% 1.8–18), occurring more frequently in the “high-risk” group. Otherwise, the incidence of SGA (small for gestational age < 10th centile) born in the two groups was not significantly different. All the above data are reported in Table 4.

### 3.1. Characteristics of the High-Risk Population for the Multi-Parameter Test

Multiparametric data processing through the software and the algorithm provided by the Fetal Medicine Foundation “Preeclampsia Predictor” recognized 20 women (3.3%) with a high risk of Early Preeclampsia and 72 women (12%) with a high risk of Late Preeclampsia. The cut-off value used to define a “high risk” pregnancy was 1:20. The results are described in Table 5. The population with high-risk tests for early PE consists of 8 cases in the low-anamnestic risk group (1.7%) and 12 cases (10.4%) in the high-anamnestic-risk group, with a statistically significant difference. A total of 72 women were at high risk of late PE. A significantly higher incidence of high-risk tests was observed in the population with high anamnestic risk (OR = 23.3; 95% CI 8.1–63.2).

The incidence of outcomes in the high-risk population for early preeclampsia is described in Table 6 (a). Overall, 83% of these developed at least one maternal adverse outcome, with a significant difference compared to low-risk cases (OR = 10; 95% CI 1.1–89.6). The difference between the two groups in the incidence of early preeclampsia and IUGR was statistically significant (OR = 47.7; 95% CI 1.7–13.1) These two complications were developed in the same case.

The differences between the two groups in the development of gestational hypertension, gestational diabetes, preterm birth, and fetal complications were not significant. In our population, the risk test for early preeclampsia showed a positive predictive value (PPV) for early PE of 21.3% and a negative predictive value (NPV) of 100%. It allowed the recognition that only 6 cases of PE occurred, compared with 27 false positives. This test also has the same predictive values for the development of IUGR, while for the development of maternal adverse outcomes, it has a PPV of 83% and NPV of 66%.

Acetylsalicylic acid intake during pregnancy was significantly higher in the group of women with a high risk of early PE (OR = 14.5; 95% CI 2.3–67.6). The incidence in the two groups is described in Table 6. Overall, 72 women were at high risk of late preeclampsia according to the result of the multiparametric test. The incidence of outcomes in the high-risk population for late preeclampsia is described in Table 7 (a). The incidence between the two groups of maternal complications was significantly higher in the population with high-risk tests (OR = 5.1; IC 95% 2.3–14.7). The incidence of gestational diabetes is also significantly higher in the high-risk population (OR = 8.1; 95% CI 3.1–32).

The differences between the two groups in the development of gestational hypertension, preeclampsia, IUGR, preterm labor, and fetal complications were not significant. With the 0% incidence of late preeclampsia in the population of our study, the PPV of the test is 0%. However, 90 cases of false positives were detected. The test did not indicate false negatives, thus showing a NPV for late PE of 100%. This test has a PPV of 65% and a NPV of 70% for the development of maternal adverse outcomes. The intake of ASA during pregnancy was significantly higher in the group of women with a high risk of late PE (OR = 15.7; 95% CI 5.1–43.4). The incidence in the two groups is described in Table 7 (b).

### 3.2. Fetal DNA

Recent studies suggest that there is a significant association between the fraction of fetal DNA and adverse outcomes during pregnancy. Contro et al. [34] showed a correlation between the fraction of fetal DNA and the complications of pregnancy, which can therefore be a marker of poor placenta development and placental dysfunction. Therefore, we evaluated the percentage of fetal DNA in our study population. A total of 101 women made fetal DNA for the screening of aneuploidies: 82 women in the group with low anamnestic risk and 19 in the group with high anamnestic risk. The results are reported in Table 8.

In our sample mean free fetal fraction DNA was significantly higher in the high-risk women (*p* = 0.02), confirming the most recent data in the literature. To justify this, the most recent scientific evidence suggests that this increase in the percentage of fetal DNA is due to abnormal placentation with episodes of placental ischemia that involve the release of abnormal quantities of necrotic and apoptotic fragments containing fetal DNA circulating in maternal blood [34]. 

Based on our data, we identified a 13% cut-off (the optimum value in terms of false positives and negatives) to discriminate women with high and low risk of PE and adverse events, with a sensitivity of 58% and specificity of 78%. Women with adverse composite maternal outcomes had a percentage of fetal DNA > 13% in 65% of cases.

## 4. Discussion

Even if the above-reported results are interesting and promising, our study must be considered a preliminary analysis, since the enrollment is still in progress. However, some conclusions can be drawn from this first analysis. The PI of the uterine arteries was not significantly different between the two study groups. This underlines how such an isolated assessment cannot represent an efficient screening method and how the calculation of the risk of developing preeclampsia must be based on a multiparametric assessment that also considers other biophysical and biochemical factors [35].

Otherwise, a significant difference has been highlighted about MAP, which is higher in the case of pregnancies at high risk based on the risk factors only, and for PAPP-A, higher in the case of low-risk pregnancies. In the literature, it has already been reported that low values of this biochemical marker are associated with a high incidence of hypertensive disorders and IUGR, whose isolated evaluation does not represent an effective screening method for the risk of developing preeclampsia [36].

The incidence of preeclampsia in our population is 1.2%. This value is in substantial agreement with the incidence of this hypertensive disorder in the general Italian population and is lower than the one found in an English study [37]. Nulliparous resulted in a higher risk for PE, probably because multiparous started cardioaspirin for the previous obstetric history.

In the study population, the positive predictive value of the risk test for late PE was 0%. There were no cases of late PE, but there were 90 false positives. The negative predictive value is 100%. This last finding, associated with the significantly higher incidence of high-risk tests for late PE in the high-anamnestic risk population suggests that this test does not provide much advantage compared to anamnestic evaluation alone.

Otherwise, the detection rate of the multiparametric screening test for early PE was very high. In fact, the test was able to identify the cases of early PE that occurred, with a low positive predictive value (21.3%). The negative predictive value of the test was instead 100%. 

Regarding the percentage of fetal DNA, according to the most recent literature data, in our series we report a statistically significant difference in the average between the low and high-risk groups.

However, the small sample size of our single-center does not yet allow us to draw definitive conclusions. Multicenter studies with even larger case studies are needed to assess the predictivity of the multiparametric test and to evaluate whether the addition of fetal DNA may have a useful implication in clinical practice.

## 5. Conclusions

In our study, we demonstrate that the multiparametric screening test for early PE performed well in identifying women at high risk of early PE, which certainly has the most severe maternal–fetal outcomes. The fact that ASA intake at low doses is significantly higher in the population with high-risk tests for both early PE and late PE suggests once again that anamnestic evaluation plays an essential role in women’s screening.

It is important to highlight that in our study, the clinical–amnestic risk stratification did not detect any of the early-onset PE cases.

All 6 cases occurred in women who had a high screen test, but only 6 out of 20 women with a high risk test developed early-onset PE. Amnestic–clinical evaluation alone is weak. Therefore, it is mandatory to recommend the PE screening in all the population to obtain the chance of cardioaspirin to prevent PE.

Even if the above-reported results are interesting and promising, our study must be considered a preliminary analysis, since the enrollment is still in progress. The small sample size of our single-center study does not yet allow us to draw definitive conclusions. Multicenter studies with even larger case studies are needed to assess the predictivity of the multiparametric test and to evaluate whether the addition of fetal DNA may have a useful implication in clinical practice.

## Figures and Tables

**Table 1:** Characteristics of the study population.

Study Population	Low Anamnestic Risk (*n* = 474)	High Anamnestic Risk (*n* = 115)	Total	Significance (*p* < 0.05)
			(*n* = 589)	
Age				NS
<20	9 (2%)	2 (2%)	11 (2%)	
20–34	336 (71%)	78 (68%)	414 (70.3%)	
35–39	94 (20%)	28 (25%)	122 (20.7%)	
>40	35 (7%)	7 (5%)	42 (7%)	
Caucasian	464 (98%)	113 (99%)	577 (98.5 %)	NS
Other	10 (2%)	2 (1%)	12 (1.5 %)	
Parity				*p* = 0.013
nulliparous	294 (62%)	29 (25%)	323 (54.8%)	
multiparous	180 (38%)	86 (75%)	266 (45.2%)	
Conception				NS
Spontaneous	465 (98%)	111 (96%)	576 (97.8%)	
Medically assisted procreation	9 (2%)	4 (4%)	13 (2.2%)	
Pregestational diabetes	4 (0.9%)	3 (2.9%)	7 (1.2%)	NS
Smoke	18 (4%)	7 (6%)	25 (4.2%)	NS

**Table 2:** Biochemical and biophysical characteristics of the study population.

Study Population	Low Anamnestic Risk (*n* = 474)	High Anamnestic Risk (*n* = 115)	Significance (*p* < 0.05)
**Pulsately Index uterine arteries ≥ 95th centile**	47 (10%)	16 (13.5%)	NS
**MAP (mean)**	81.8 ± 7.1	96.2 ± 9.4	*p* = 0.032
**PAPP-A**	3671.2 ± 1995.3	1732.5 ± 1478.2	*p* = 0.002
**PIGF**	31.7 ± 9.8	28.9 ± 6.7	NS

**Table 3:** Anamnestic risk factors in the study population.

Anamnestic Risk Factors	High Anamnestic Risk (*n* = 115)
Obesity	95 (83%)
Chronic hypertension	11 (10%)
Previous preeclampsia	14 (12%)
Previous Intrauterine fetal death	5 (5%)
Prior intrauterin growth restriction	6 (6%)

**Table 4:** Adverse outcomes in the study population.

Population	Low Anamnestic Risk (*n* = 407)	High Anamnestic Risk (*n* = 91)	Total (*n* = 498)	Significance *p* < 0.05
Miscarriage	9 (2.2%)	3 (3%)	12 (2.4%)	NS
Gestational diabetes	48 (12%)	35 (38.8%)	83 (17%)	*p* < 0.0001
Gestational hypertension	8 (2%)	7(8.1%)	15 (3.01%)	NS
Preeclampsia	6 (1.4%)	0 (0%)	6 (1.2%)	NS
Hypertensive disorders	20 (5%)	8 (9%)	28 (5.6%)	NS
Intrauterine Growth Restriction	1 (0.2%)	0%	1 (0.2%)	NS
Intrauterine fetal death	0 (0%)	0 (0%)	0 (0%)	NS
Hemolysis, Elevated Liver enzymes and Low Platelets	0 (0%)	0 (0%)	0 (0%)	NS
Placental Abruption	0 (0%)	0 (0%)	0 (0%)	NS
Premature rupture of membranes	0 (0%)	3 (3%)	3 (0.6%)	NS
Cholestasis	17 (4.1%)	0 (0%)	17 (3.4%)	NS
Neonatal weight > 2500 g	374 (92%)	85 (93%)	459 (92%)	NS
Neonatal weight ≤ 10° (Small for Gestational Age)	28 (7%)	6 (6%)	34 (6.8%)	NS
Neonatal weight centile ≥ 90° (Large for Gestational Age)	41 (10%)	27 (30%)	68 (13.7%)	*p* = 0.02
Apgar < 7 to 10′	0 (0%)	0 (0%)	0 (0%)	NS
Preterm birth	8 (2%)	2 (2%)	10 (2%)	NS
Labor				*p* = 0.0002
No labor	65 (16%)	14 (15%)	79 (15.8%)	
Spontaneous	274 (67.4%)	58 (64%)	332 (66.7%)	
Induction	68 (16.6%)	19 (21%)	87 (17.4%)	
Type of deliver				NS
Vaginal Birth	309 (76%)	67 (74%)	376 (75.5%)	
Cesarean birth	98 (24%)	24 (26%)	122 (24.5%)	

**Table 5:** Results of the multiparametric test in the study population.

Study Population	Low Anamnestic Risk (*n* = 474)		High Anamnestic Risk (*n* = 115)	
	Early PE	Late PE	Early PE	Late PE
High-risk test (≥1:20)	**8 (1.7%)**	**22 (4.6%)**	**12 (10.4%)**	**50 (43.5%)**
Low-risk test (<1:20)	**466 (98.3%)**	**452 (95.3%)**	**103 (89.6%)**	**65 (56.5%)**

**Table 6:** (a) Incidence of outcomes in the high-risk population for early PE. (b) Incidence of ASA intake based on early PE risk.

(a)					
Risk Test for Early PE	Low Risk (*n* = 478)	High Risk (*n* = 20)	Total (*n* = 498)	False Positive (*n* = 498)	False Negative (*n* = 498)
**Preeclampsia**	**0 (0%)**	**6 (30%)**	**6 (1.2%)**	**27 (5.4%)**	**0 (0%)**
Gestational hypertension	**22 (4.7%)**	**1 (5%)**	**15**	**27 (5.4%)**	**22 (4.3%)**
IUGR	**0 (0%)**	**1 (5%)**	**1 (0.2%)**	**27 (5.4%)**	**0 (0%)**
Gestational diabetes	**73 (15%)**	**10 (50%)**	**83 (16.6%)**	**16 (3.2%)**	**101 (20.4%)**
Preterm birth	**6 (1.2%)**	**4 (17%)**	**10 (2%)**	**27 (5.4%)**	**5 (1.1%)**
Maternal composite outcomes	**165 (34.5%)**	**17 (83%)**	**182 (36.5%)**	**5 (1.1%)**	**155 (31.2%)**
Fetal composite outcomes	**19 (3.9%)**	**4 (16.7%)**	**23 (4.6%)**	**27 (5.4%)**	**10 (2.2%)**
**(b)**					
**Risk Test for Early PE**	**Low Risk** **(*n* = 478)**	**High Risk** **(*n* = 20)**	**Total** **(*n* = 498)**	**Significance** ***p* < 0.05**	
Assumption of ASA	62 (13%)	14 (72%)	76 (15.3%)	*p* = 0.02	

**Table 7:** (a) Incidence of outcomes based on the risk of late PE. (b) Incidence of ASA intake based on late PE risk.

(a)					
Risk Test for Late PE	Low Risk (*n* = 397)	High Risk (*n* = 101)	Total (*n* = 498)	False Positive (*n* = 498)	False Negative (*n* = 498)
Preeclampsia	**0 (0%)**	**0 (0%)**	**0 (0%)**	**90 (18%)**	**0 (0%)**
Gestational hypertension	**9 (2.3%)**	**6 (6%)**	**15 (3%)**	**40 (17.2%)**	**21 (4.3%)**
Hypertensive disorders	**22 (5.5%)**	**6 (6%)**	**28 (5.6%)**	**40 (17.2%)**	**21 (4.3%)**
IUGR	**1 (0.2%)**	**0 (0%)**	**1 (0.2%)**	**90 (18%)**	**5 (1.1%)**
Gestational diabetes	**22 (5.5%)**	**61 (60%)**	**83 (16.6%)**	**37 (7.5%)**	**64 (12.9%)**
Preterm birth	**4 (1%)**	**6 (6%)**	**10 (2%)**	**84 (17.2%)**	**5 (1.1%)**
Maternal composite outcomes	**116 (29.2%)**	**66 (65%)**	**182 (36.5%)**	**32 (6.5%)**	**123 (24.7%)**
Fetal composite outcomes	**23 (5.8%)**	**0 (0%)**	**23 (4.6%)**	**91 (18.3%)**	**16 (3.2%)**
Assumption of ASA	11	65	76 (15.3%)	*p* = 0.02	
**(b)**					
**Risk Test for Late PE**	**Low Risk** **(*n* = 397)**	**High Risk** **(*n* = 101)**	**Total** (***n* = 498)**	**False Positive** **(*n* = 498)**	
Assumption of ASA	11	65	76 (15.3%)	*p* = 0.02	

**Table 8:** Percentage of fetal DNA fraction in the study sample.

	Low Risk (*n* = 82)	High Risk (*n* = 19)	Significance *p* < 0.05
% fetal fraction, mean ± SD	8.7 ± 2.1	14.2 ± 3.2	*p* = 0.02

## Data Availability

Not applicable.
